# Development and validation of a multivariable prediction model for mediastinitis poststernotomy

**DOI:** 10.1186/2197-425X-3-S1-A955

**Published:** 2015-10-01

**Authors:** M Nieto, M Sanchez, B Busto, E Morales, I Garcia, C Manuel, L Cano, C Fernandez

**Affiliations:** Hospital Clinico San Carlos, Intensive Care, Madrid, Spain; Hospital Clinico San Carlos, Madrid, Spain; Hospital Clinico San Carlos, Cardiac Surgery, Madrid, Spain; Hospital Clinico San Carlos, Epidemiology Unit, Madrid, Spain

## Introduction

Mediastinitis is a severe complication of major heart surgery (MHS). Scoring systems constitute a very useful tool for risk assessment and to establish appropriate prevention strategies.

## Objectives

To design a predictive model of mediastinitis for bedside estimation and to develop and validate a risk score for stratification. To compare our model with other indices.

## Methods

Data of 4526 patients admitted after MHS to our Cardiovascular ICU (January 2005,-June 2011) were prospectively collected. After exclusion of 655 patients, 3970 were analyzed to identify risk factors for mediastinitis. A model was generated by logistic regression analysis in a subgroup of 2618 randomly selected patients and validated in a second cohort of 1352. The discriminatory power was evaluated by area under the receiver operating characteristic curves (AUC-ROC). Calibration was done by the Hosmer-Lemeshow test. The mediastinitis-score (“MED-Score”) was created using the points estimate for each variable, with beta coefficients of the final model. For stratification three risk levels were generated.

We assessed the ability of “MED-Score, Euroscore (ES)1, and the Society of Thoracic Surgeons score (STS)2 to predict mediastinitis using ROC curves comparison (Mann-Whitney). Concordance of risk stratification by scoring systems was evaluated using weighted Kappa index. We used SPSS vs15.0. and STATA 11.0. The outcome “mediastinitis” was defined according to CDC criteria.

## Results

94 (2.4%) patients developed mediastinitis. Four preoperative (age >70, COPD, BMI>30 and antiplatelet therapy) and 3 perioperative predictors of mediastinitis (ischemia time, emergency reoperation and prolonged intubation) were identified and included in the logistic model, which accurately predicted outcome (AUC.ROC 0.80). Hosmer-Lemeshow test p = 0.46. The AUC for preoperative Society of Thoracic Surgeons (STS), and combined STS, logEuroScore had a lower discrimination power than MED-Score. This difference was statistically significant. Concordance of risk stratification by the different scoring systems was poor (Kappa< 0.20)

## Conclusions

The predictive model based on 7 factors showed excellent predictive power. Our score provides a simple tool for stratification of MHS patients. High-risk patients may be targeted for prevention strategies. In our cohort of patient the STS scores and logES are suboptimal predictors of mediastinitis

**Figure 1:**
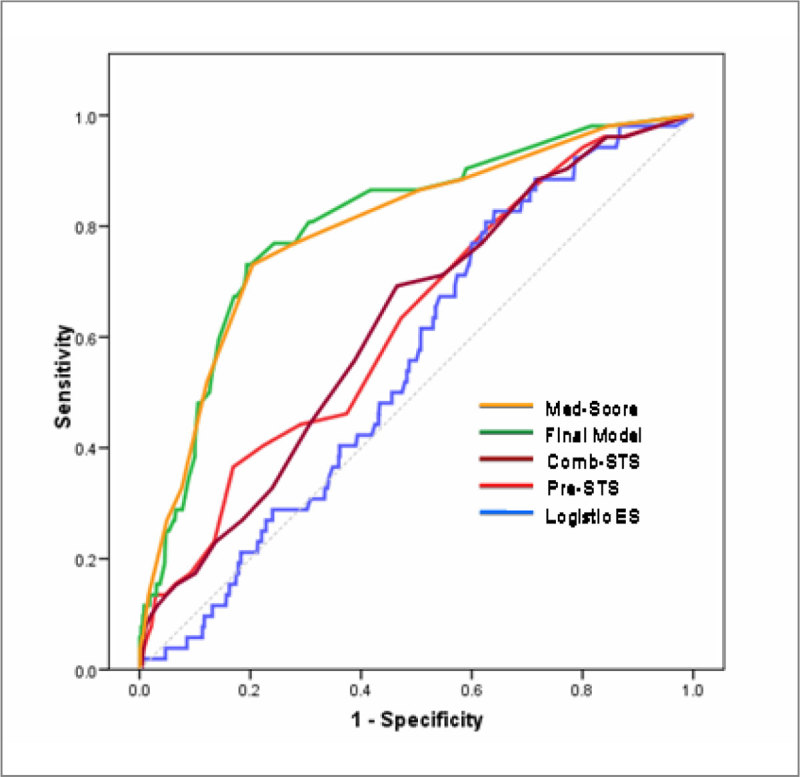
**Discrimination models**.
